# Prioritization strategies in clinical practice guidelines development: a pilot study

**DOI:** 10.1186/1478-4505-8-7

**Published:** 2010-03-06

**Authors:** Ludovic Reveiz, Diana R Tellez, Juan S Castillo, Paola A Mosquera, Marcela Torres, Luis G Cuervo, Andres F Cardona, Rodrigo Pardo

**Affiliations:** 1 Clinical Research Institute and Health Technology Assessment Unit, National University, Bogota, Colombia; 2 Foundation for Clinical and Translational Research in Cancer (FICMAC), ONCOLGroup, Bogota, Colombia; 3 Research Promotion and Development, Pan American Health Organization, Washington, USA; 4 Clinical and Translational Oncology Group, Institute of Oncology, Fundación Santa Fe de Bogotá, Bogotá, Colombia

## Abstract

**Objective:**

Few methodological studies address the prioritization of clinical topics for the development of Clinical Practice Guidelines (CPGs). The aim of this study was to validate a methodology for Priority Determination of Topics (PDT) of CPGs.

**Methods and results:**

Firstly, we developed an instrument for PDT with 41 criteria that were grouped under 10 domains, based on a comprehensive systematic search. Secondly, we performed a survey of stakeholders involved in CPGs development, and end users of guidelines, using the instrument. Thirdly, a pilot testing of the PDT procedure was performed in order to choose 10 guideline topics among 34 proposed projects; using a multi-criteria analysis approach, we validated a mechanism that followed five stages: determination of the composition of groups, item/domain scoring, weights determination, quality of the information used to support judgments, and finally, topic selection. Participants first scored the importance of each domain, after which four different weighting procedures were calculated (including the survey results). The process of weighting was determined by correlating the data between them. We also reported the quality of evidence used for PDT. Finally, we provided a qualitative analysis of the process. The main domains used to support judgement, having higher quality scores and weightings, were feasibility, disease burden, implementation and information needs. Other important domains such as user preferences, adverse events, potential for health promotion, social effects, and economic impact had lower relevance for clinicians. Criteria for prioritization were mainly judged through professional experience, while good quality information was only used in 15% of cases.

**Conclusion:**

The main advantages of the proposed methodology are supported by the use of a systematic approach to identify, score and weight guideline topics selection, limiting or exposing the influence of personal biases. However, the methodology was complex and included a number of quantitative and qualitative approaches reflecting the difficulties of the prioritization process.

## Introduction

The need to set priorities for developing Clinical Practice Guidelines (CPGs) has been recognized by several CPG developers, in part due to the rapid development of medical technology and finite resources of organizations [1,2]. Although choosing which topic to address in CPGs (prioritization) is a process that is not always followed by standardized processes, explicit and/or implicit choices about how to allocate funds and staff time to particular projects must be made by organizations [1-3].

Prioritization to Determine Topics (PDT) for CPGs is frequently influenced by the availability of human and financial resources. PDT also varies according to specific needs of sponsors, such as health care organizations and the different hierarchical levels within the health research system [3,4]. A methodology that uses explicit criteria and a systematic approach for PDT may facilitate the implementation of an open, verifiable and reproducible PDT [5]. Although qualitative and quantitative methods have been used in PDT, there are few published studies addressing the group selection and methodology that has been followed when picking clinical topics for the development of CPGs [5-8].

We used an explicit methodology to prioritize topics for CPGs in order to evaluate the topics identified with this methodology and see if they were, in fact, considered high priority.

## Methods

**Figure 1:**
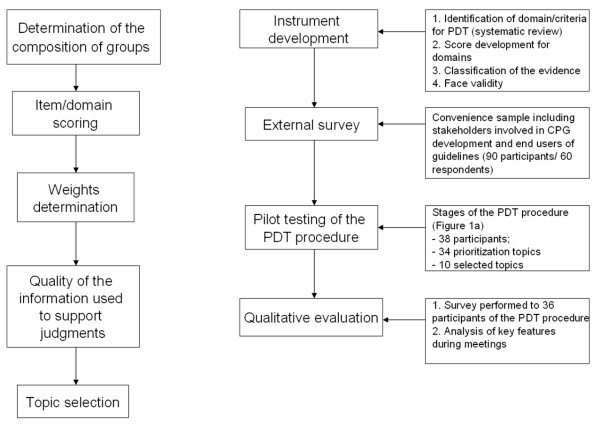
**Stages of the PDT procedure (left side) and methodological approaches used for validating the process (right side)**.

The primary aim of this study was to develop and validate an explicit methodology for PDT of CPGs within the setting of a developing country. We used a number of different approaches to develop and assess the PDT process: instrument development; an external survey; a pilot testing of the PDT procedure and; a qualitative evaluation. The PDT procedure integrated quantitative and qualitative data from different sources. A summary of the stages of the PDT procedure as well as the methodological approaches used for validating the process are shows in Figure 1a and 1b respectively.

### Instrument development

We developed a formal instrument for prioritizing guideline topics with the following characteristics: domain/criteria importance; quality of the supporting evidence for scoring each domain; availability of supporting bibliography; additional domains or criteria not listed in the tool.

#### Identification of domain/criteria for PDT

To identify criteria for PDT and summarize knowledge, methods and strategies used for PDT in CPGs development, we conducted a systematic search in the following databases: MEDLINE (PUBMED 1966 to July 2008), The Cochrane Library (Issue 2, 2008) and LILACS (1982 to July 2008). Unfortunately, we did not have access to CINAHL, which frequently contains useful studies. The search strategy was structured and adapted according to each electronic database (Appendix 1). Besides, we reviewed the websites of several recognized governmental and nongovernmental organizations that produce CPGs, including the National Institute for Clinical Excellence http://www.nice.org.uk, the Scottish Intercollegiate Guidelines Network http://www.sign.ac.uk, the World Health Organization [WHO; http://www.who.int], the New Zealand Guidelines Group http://www.nzgg.org.nz/, The Canadian Medical Association InfoBase, Guidelines Advisory Committee http://www.gacguidelines.ca/ National Guideline Clearinghouse http://www.guideline.gov/resources/guideline_resources.aspx, and the G-I-N database http://www.g-i-n.net. The search included observational, descriptive, comparative, interventional studies and reports published in CPG clearinghouses. A number of reports were identified and analyzed [5-7,9-17]; however only one methodological paper on CPG prioritization was identified [5]. Forty-one items were determined and categorized into 10 domains, established a priori (Table 1) related with CPGs and other research prioritization papers [5-7,9-17] and websites of CPG producers.

**Table 1:** Criteria for setting priorities according to their assessed importance by participants and external stakeholders.

Domain	Items	Mean participants' weighted score (SD)
Disease Burden	- Disease/Condition incidence or prevalence - High risk impact of disease/condition in the health system - High frequency of risk factors associated with the disease/condition - High frequency of avoidable risk factors associated with the disease/condition	80,8 (18,2)

Information needs in the Health Sector	- Information needs within the Institution/Organization - Current controversy about topic importance - High importance of new methods and technology assessment - Fast diffusion of non-assessed technologies, availability of resources and sufficient time for technologies implementation - Country health priorities in agreement with CPG's needs - High impact on national health system	74,7 (18,1)

Feasibility on development and implementation	- Feasibility on recommendations development which will improve health outcomes and cost - Is the proposal politically feasible? - Does it belong to priority health areas according to government policies? - Feasibility in implementation; will not require an excessive amount of resources and will not present important barriers to implement changes - Will reduce inequities when implemented - Will require education to training professionals - Does the proposed topic include the participation of multiple departments, institutions, organizations, etc?	72,7 (17,1)

Effectiveness	- Availability of effective methods shown by methodologically adequate studies. - Certainty about effectiveness of assessed interventions and technologies - Potential impact of CPG	71,2 (20,7)

Economic impact on the health system	- Economic effects on health system (cost of an individual patient is high during diagnosis or therapeutic process) - Disease/Condition associated with iatrogenic interventions that are significantly high in cost.	69,8 (24,0)

Clinical Practice Variation	- Current evidence is insufficient for disease control in the population - Lack of high quality CPGs - Availability of high volume of evidence regarding the CPG topic - Evidence of inappropriate use of available technologies used in the treatment of condition (iatrogenic) - Conditions/diseases where effective treatments could reduce mortality or morbidity - Evidence of disagreements between current treatment and literature recommendations.	68,0 (22,1)

Other social effects/Equity	- Absenteeism from work or school, inability to work, inequities in access to health services - Will the service be available to anyone who requires it? - Will this CPG have a positive or negative impact on minorities' access to health services? - Will the CPG increase health service access to those affected by the condition?	67,2 (25,7)

User Preferences	- High patient demand or interest - Concerns about patients' quality of life - Feasibility of patient empowerment - High acceptability of the topic between the general public and professionals affected by the use of the CPG.	64,9 (22,3)

Adverse events	- Possibility of adverse events - Possibility of serious adverse events - Disease/condition associated with high incidence of adverse events or sequels	57,1 (28,0)

Health Promotion and Disease Prevention	- Feasibility of prevention between patients with risk factors - Are there specific activities of health promotion, disease prevention, early diagnosis or treatment? Have all of them shown a reduction in disease burden?	56,4 (32,3)

* The maximum score was 100 and the minimum was 0

#### Scoring each domain

According to its perceived importance, each of the ten domains was scored independently by evaluators according to a preference scale from 0 to 100.

#### Classification of the evidence

The quality of the information used to support each judgment for every prioritized topic (for each of the 41 items in the instrument) was categorized as follows: good quality information; moderate quality information; bad quality information; professional experience only; no information or not applicable.

#### Face validity

Face validity of the instrument was evaluated by four epidemiologists, a public health physician, a psychologist, and a health administrator. Evaluators were in agreement that the instrument appeared sound and relevant with a logical tie between the purpose and the criteria, without areas of omission. Questions and instructions were considered clear, unambiguous, logical, grammatically correct and free of excess wording. Choice options were also judged to be appropriate for the instrument and clearly defined. An English version of the instrument is available as an additional file [see Additional file 1].

### External questionnaire

Because the judgment about the importance of a domain may vary according to several factors, such as the proposed topic or the background of the decision makers, we surveyed a number of stakeholders involved in CPG development, in addition to end users of guidelines. The objective of the survey was to collect data in order to compare different rating procedures for the prioritization methodology (see below).

The convenience surveyed sample consisted of 90 people, comprising external stakeholders involved in CPG, and end users of guidelines (patients, health care providers including clinical staff, government officials, representatives from the pharmaceutical industry and private health care managers and academic researchers) that were identified from different sources (research institution lists, guideline developers and stakeholders from Colombia found in Google Scholar, colleagues etc.) and who had an active electronic address. As electronic surveys usually have lower rate response [18], we expected that at least 50% of surveyed reply.

Each participant was sent a survey via an email containing the instrument, as well as instructions for rating each domain according to its relevance for PDT in CPGs. Up to four reminders was sent. The main outcome was the rating score for each dominium (range 0 to 100). No particular topic for CPG development was used at this stage. We also asked participants about additional domains or criteria not listed in the tool, as well as additional commentaries on the instrument. We received 60 responses representing all different types of participants. There was no significant difference in the proportion of the different types of respondents (data not shown). No substantial modifications to the instrument were suggested. Results on weights for each domain were incorporated in the rating procedures (see the "*weighting the domains*" section).

### Pilot testing of the PDT procedure

#### 1. Design

Taking into account the fact that ten CPG topics should be selected from the process, the final objective of the study was to develop and validate an explicit methodology for PDT of CPGs. We also tested different strategies for weighting the domains. The PDT model adopted in this study is based on a multi-criteria analysis approach which contains five stages [6,7]:

##### 1.1 Composition of the guideline development team

The composition of the CPG development panel may have an impact on the content of the guideline recommendations. Empirical evidence shows that panels with more than 12 members may have better judgment [8-12]. Bearing in mind that there are technical and administrative issues that are important to PDT, we worked on two distinct groups: thematic and administrative. The thematic group included three experts on the field and one methodological consultant. Experts from the thematic group included 30 representatives from nine departments of the Medical School and one from the Nursing School of the National University of Colombia (cardiology, gynecology, anesthesia, surgery, pediatrics, radiology (2 teams), nursing, endocrinology and neurology departments). The administrative teams included an expert in psychology, two members from the hospital board and a project manager. Finally, all 38 participants were involved in the methodology for PDT of 10 different CPGs.

Following a workshop which had explained the methodology for PDT to the team members, the thematic team suggested three to five clinical topics that could potentially be selected for developing a CPG. Subsequently, participants used the instrument to score the importance of domains for each proposed topic. A web-based tool was developed to allow participants to communicate and track the guideline development process. The selection of topics was completed three weeks later in a second meeting.

##### 1.2 Scoring domains

As mentioned before, 41 items were determined and categorized into 10 domains (Table 1). To allow participants to use the predefined criteria in categorizing information into the various domains, we developed a guiding document that contained a list of domains in alphabetical order (in Spanish). Participants were asked to score each proposed topic for its importance, rate the quality of the supporting evidence, the availability of supporting bibliography, and space to suggest additional domains or items not listed in the tool (see Annex1).

##### 1.3 Weighting the domains

We followed the methodology proposed by Oortwijn et al. [5] by weighting each domain and then using standardized scores according to this equation:

S = score of each domain

Min = Lowest score allocated by participants

Max = Highest score allocated by participants

We first applied a "non-weighted procedure" in which total scores (TS) were the sum of each domain (without categories adjustment):

TS = A + B + C + D + E + F + G + H + I + J

Subsequently, domains were grouped by disease, social and economic considerations (Table 2).

**Table 2:** Groups of domains according to their potential effects on health, cost, feasibility and health policy.

Type I Criteria with potential effects on health	Disease Burden
	Effectiveness
	Adverse Events
	Health Promotion
Type II Criteria with potential effects on cost and feasibility	Feasibility on development and implementation
	Economic impact on health system

Type III Additional aspects relevant for health policy	Information needs within the health sector
	Other social effects/equity
	User preferences
	Clinical practice variation

Classification of domain for the weighting strategies: A: Disease Burden; B: Information needs in the Health Sector; C: Effectiveness; D: Adverse events; E: Feasibility on development and implementation; F: Economic impact on the health system; G: Other social effects/Equity; H: Health Promotion and Disease Prevention; I: User Preferences; J: Clinical Practice Variation

A second weighting strategy followed the "equal weights procedure" adjustment:

TS = (A + C + D + H) + ((E + F)*2) + (B + G + I + J)

The third weighting strategy was a "different weights procedure" in which each type was adjusted in accordance with the final Oortwijn's weighting strategy [5]:

TS = (A + C + D + H) + ((E + F)*0.5) + (B + G + I + J)

We developed the fourth weighting strategy to adjust the type II and III criteria weights according to the "reported preferences" of CPG users' external survey:

TS = (A + C + D + H) + ((B + G + I + J) *0.5) + ((E + F)*0.25)

We sorted scores in ascending order and calculated their distribution by percentiles to generate strata by topic relevance, grading topics in the following categories: low relevance for those in the first quartile; intermediate relevance for those in the two interquartile ranges; and high relevance for the upper quartile. We compared the relevance qualification obtained for the ten selected topics in each one of the weighting methods.

##### 1.4 Classification of evidence used for PDT

Participants rated the quality of the information used to support their judgments for each of the 41 items on the form, as previously described. Professionals with methodological and scientific literature search expertise (epidemiologists and information specialists) assisted the teams when requested and provided relevant references as needed.

##### 1.5 Selection of topics

Depending on which teams were weighing the topics, rankings were categorized by (1) thematic team, (2) methodological team, or (3) administrative team. Rankings listed topics as having low, intermediate or high relevance and then a final selection was made during a consensus meeting in which the pre-defined tools were used.

#### 2. Data collection

After being informed of the purpose and nature of the procedure, requirements and responsibilities of participants, an Excel sheet file was sent to all participants by email. Trained evaluators assisted as needed with completion of the information. Data was collected in an Excel file and analyzed using Stata 8.0.

#### 3. Statistical analysis of data

To evaluate different strategies for weighting the domains, we tested the null two-sided hypothesis with a 90% power and 5% significance for a correlation effect of 0.5. Therefore, we needed a sample size of 31 participants [Ho: ρ_0 _= 0; 90% power; 5% level (two-sided)] using the methods described by Kraemer [19] for nonparametric tests. We estimated means, medians and ranks of weightings and quality scores by topic (items). We used the Kruskal-Wallis test to examine differences between the ranks of the median weightings among selected topics or non-selected topics. We considered P < 0.05 (two-sided) to be significant for each test. We hypothesized that particular domains are used for decision-making. Principal components analysis of the partial correlation matrix (Varimax rotation) was used to identify underlying factors.

### Qualitative evaluation

#### 1. Participants and materials

36 of the 38 participants in the prioritization of topics received by email an open-ended questionnaire inquiring about their experience with the prioritization process. Up to two reminders were sent. Data from respondents were collected by a psychologist for analysis.

#### 2. Procedures

The questionnaire was centered on the following areas:

- Advantages, limitations and barriers of using the instrument

- Facilitators, limitations and barriers of the PDT procedure

- Power relation within groups

- Others

This was complemented with direct observation methods to identify their reactions to the process during the two meetings. The sessions were facilitated by a guideline methodologist and co-moderated by the psychologist.

#### 3. Data analysis

Methods used for data analysis included speech analysis (written data) and triangulation of arguments (verbal data); both were led by a psychologist (PM). Three steps for analysis were followed:

##### Step 1. A content analysis of the text of the survey was performed as follows [20]

1. Responses were thoroughly read in extensor several times, aiming for a global understanding of the content.

2. Data was categorized under different themes.

3. Main themes were condensed. Inferences were drawn collectively by careful reading of the text under each theme.

4. Deviant cases of participants' statements were identified.

##### Step 2. Analysis of key features during meetings

The group sessions followed the following pattern: i) welcome and self-introduction, ii) explanation of the instrument and PDT procedure, iii) general discussion proper, iv) organization of the ten different groups representing nine departments of the Medical School and one from the Nursing School in order to generate a discussion of the particular topics, v) general discussion, allowing participants to speak if they had anything further to say, and vi) appreciation for participation.

Each group also had a moderator (from the methodological team) and an observer (psychologist). As only one experienced psychologist was available, working groups were evaluated at different times during the sessions. Each group discussion was reorganized under the identified themes. The prioritization instrument was presented during the first meeting. Topic selection was performed during the second meeting.

##### Step 3. Correspondence between main themes of the survey of participants and key features of meetings

Results from content analysis of the survey of participants and content analysis of key features of meetings were brought together and corresponding patterns were looked for. Triangulation was used to increase the validity and reliability of the study in order to identify what remains constant, or is common to all the different perspectives [21,22]. To establish communication functions we made an internal analysis of texts and identified the use of terms, and content. The texts were read with care and re-read to identify key codes. Finally, we analyzed the properties of categories and explored connections between them. Interpretations were discussed between authors, and after analyses, concordance was reached. Resulting analysis was contrasted with those obtained from the quantitative study [23].

The guideline development project was approved by the ethics board committee of the National University of Colombia. Written informed consent was not sought in the internet-based survey but informed consent of participants was implied because access to the questionnaire was restricted to persons approached by e-mail explaining the study.

## Results

### Assignation of weight to identified criteria

**Figure 2:**
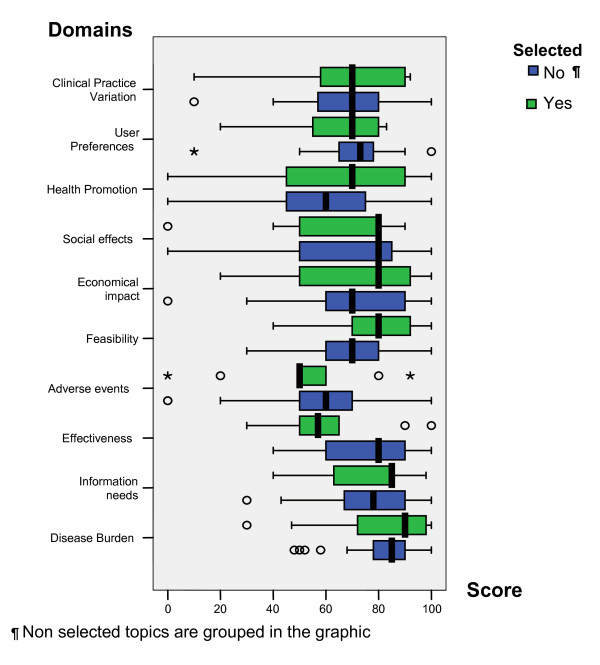
**Participants' score of relevance for each domain in the 34 evaluated topics and according to whether or not they were selected**.

Overall, 34 topics were proposed, of which 10 were chosen. Mean values for each domain are summarized in Table 1; the range is from 56.35 to 80.79. Teams considered disease burden, needs of information, feasibility and effectiveness as the most relevant domains, while health promotion and adverse events were considered the least important. We found no statistical differences in weighting domains for each topic (K. wallis p > 0.05) [Figure 2]. In addition, no significant differences related with the four weighting systems were found when comparing selected and non-selected topics [Table 3].

**Table 3:** Comparison of selected topic relevance according to different weighting type procedures.

Topic relevance	*Non-weighted procedure*	*Equal weights procedure*	*Different weights procedure*	*Weighted based on the external survey*
*High relevance*	2, 3	2, 3	2, 3	2, 3, 8

*Intermediate relevance*	4, 5, 6, 7, 8	4, 5, 6, 7, 8, 9	4, 5, 6, 7, 8	4, 5, 6, 7

*Low relevance*	1, 9, 10	1, 10	1, 9, 10	1, 9, 10

1. Cardiology; 2. Gynecology; 3. Anesthesia; 4. Surgery; 5. Pediatrics; 6. Radiology A; 7. Nursing; 8. Radiology B; 9. Endocrinology; 10. Neurology.

### Classification of evidence used for PDT

The report of the quality of the information used to judge the 41 items for each of the 34 prioritization topics by every thematic team was mainly based on professional experience (32%); 15% and 30% of the answers were based on good and moderate quality information, respectively, according to the respondents. The main domains used to support judgment were feasibility, effectiveness and disease burden, which had higher quality scores and weightings. Other relevant domains, such as user preferences, social effects, adverse events and economic impact were less used and had lower quality report scores. The overall quality scores by topic are summarized in Table 3. Finally, we found no statistical differences in quality scores by topic among selected or non-selected topics (K. wallis p > 0.05)

### Factor Analysis

A factor analysis (explaining variance 92%) identified three underlying components or factors: the first one comprised clinical practice variation, adverse events, information needs, effectiveness and disease burden; the second factor included the social effects and user preferences; and the third factor comprised the economic impact and health promotion. The main domains used to support judgment having higher quality scores and weightings were feasibility and disease burden. Other important domains, such as user preferences, social effects and economic impact, had lower importance.

### Qualitative analysis of PDT

All of the participants responded to the survey. Main issues identified during PDT were the delays in establishing the teams, different working dynamics inside the groups, the lack of familiarity with the use of virtual tools, the short time available for literature searches, and the lack of access to a number of full text articles. Other factors, such as the management of hierarchies, individual preferences and the power relations within the groups, emerged in the participants' comments and were recognized as underlying factors that influenced the decision making of groups.

Several factors were identified as facilitators for the prioritization process: the development of a formal instrument and instructive, the continued support of the methodological teams, and the experience of the experts on the proposed topics.

Participants suggested that future PDT processes increase the teams' awareness of the importance of reducing the influence of internal factors such as individual preferences and management of power relations in decision making within each thematic group.

## Discussion

### Major findings

The priority setting process is usually complex, having both quantitative and qualitative components [3-5]. To the best of our knowledge, this is the first study that formally incorporates the report of the quality of the information for every domain in the prioritization of topics for CPGs. By developing a multi-criteria analysis approach we used explicit items and transparent processes to select topics for CPGs.

Our findings allowed us to identify that burden of disease, feasibility, and information needs were the most relevant domains for the participants' guideline topic selection in our local settings. Adverse events and health promotion had the lowest scores, probably suggesting the low awareness that the experts have concerning these domains. These findings should encourage CPG developers to increase the awareness of the domains that increase patient safety. Our study makes it clear that although most proposals were considered because of their priority importance, other topics were chosen according to individual preference of the thematic team, rather than as a result of a detailed search and evaluation of the existing evidence for each topic. This poses a challenge to methodologist researchers on how to manage political weights versus scientific weights in making the final decision on prioritized CPG topics.

The use of a formal procedure generates data which can be used with assertiveness and with results that provide an initial ranking list that helps to shape the decision-making process. Although we believed that the relevance of weighting could be crucial in determining the possible scores and helps to distinguish among the most important topics in the prioritization process, our study found no difference between the four tested models.

The inclusion of the point of view of administrative and methodology teams in the decision-making process added a new component that increases the validity of the chosen topics. Our results are similar to the findings from Oortwijn et al. [5] who found that different ratings of research proposals can have different policy relevance, and that it was also necessary to include the point of view of the organization for which the CPG is developed. However, the narrow range of scoring found for each domain was probably due to the lack of knowledge in some particular domains or to the low discriminatory power in those domains.

One-third of the items were scored according to professional experience and only 13% were supported with good quality literature as reported by the participants. Participants frequently reported good quality of the information for effectiveness and feasibility domains. Although the lack of time to generate information for all the domains may have influenced findings, it could also be due to the lack of skills in performing advanced search strategies, the lack of evidence in some particular topics and domains, as well as the lack of awareness in a number of domains, as previously mentioned. Even if the thematic teams had the possibility of asking a methodologist to look for evidence, most of them did not seek assistance. This is evidence of the lack of quantity and quality that the information can have; routine decisions are usually taken by clinical appraisers, and there is a necessity to deepen the formation in evidence-based medicine to support clinical decisions.

The prioritization method can be considered a feasible and transparent approach toward setting priorities in our context; however, the process is new and there is still room for improvement. In the future, we will consider developing strategies to ensure representativeness of the experts, to evaluate the methodology by comparing it to the results of other methods and to evaluate the reports used to support the decisions and to socialize our findings with a wider audience.

### Limitations of our study approach

This study has several limitations; the methodology was quite complex and included a number of quantitative and qualitative approaches that reflects the difficulties of the usual process of prioritization. Although, we intended to provide a systematic approach to establish criteria for PDT, there were inherent difficulties and limitations. The sample selection of the external survey was based on convenience sampling, which has limitations for ensuring external validity. The composition and structure of the groups may have an impact in the individual and group decision making. Groups vary according to their size, leadership styles, training, external support, roles and rules among others. The way our group was established may have some difference with other guideline developers, limiting the external validity of our study. Although we defined the scale for rating the quality of the studies used to support the judgment of the thematic teams, we did not use a formal tool and we did not check for the accuracy of the interpretation of the evidence provided by the participants.

Several studies have identified certain criteria, such as disease burden, as the most important factors in the decision-making process [14-17]. Although the findings might have been biased by the choice of the thematic team, we included different points of views to control and socialize their topic selections, including an administrative team and a methodological consultant team. In addition, this approach contributed toward identifying the points of agreement and the points of controversy; the discussion was therefore focused on the relevant issues of the topic selection. Although the final decision of the selection of topics had to be taken by the thematic teams, our approach led us to a short list of topics in an open and verifiable process.

## Conclusions

The results of this study draw attention to important issues for policy-makers. Firstly, the selection of the conditions in the decision-making process includes several criteria at different levels. Secondly, not all criteria are equally important for clinicians. The main advantages of the proposed methodology are that it uses a systematic approach to identify, score and weight guideline topics, limiting or exposing the influence of personal biases. In addition, the prioritization process included the contributions of each stakeholder, in an effort to prevent the possibility of a few stakeholders taking over the process by producing biased topics for CPG. It also clarified the process with the use of quantitative measures and qualitative techniques, evaluating different aspects of one specific topic which helped to identify the strengths or weakness of the proposed alternative. We acknowledge there is a need for more epidemiological research in our context, in order to provide more accurate information to the guideline developers so they can weigh up the criteria in a more precise way for the selected topics.

## Appendix 1. Search strategy for PUBMED

("Guideline " [Publication Type] OR "Guidelines as Topic" [Mesh] OR "Practice Guideline " [Publication Type] OR clinical practice guideline* [tw] OR public health guideline* [tw]) AND (Health Priorities [mh] OR Health Priorit* [tw] OR priorit* setting [tw] OR (priorit* method* [tw]) OR (priorit* technique* [tw]) OR (priorit* strateg* [tw])).

## Declaration of competing interests

The authors declare that they have no competing interests.

LGC has contributed to this study in a personal capacity and in his spare time. The Pan American Health Organization (PAHO) does not assume responsibility for the statements contained therein.

## Authors' contributions

All authors participated in the design of the study, in addition to reading and approving the final manuscript. LR, JSC and DT conceived the study, and participated in its design and coordination and draft the manuscript. JSC performed the statistical analysis. MT, PM, PO, DT, LGC and RP helped to draft the manuscript. All authors read and approved the final manuscript.

## Supplementary Material

Additional file 1 **Instrument for prioritizing guideline topics**. the instrument includes characteristics such as the domain/criteria importance; the quality of the supporting evidence for scoring each domain; the availability of supporting bibliography. Click here for file
